# Antibiotic resistance among the Lahu hill tribe people, northern Thailand: a cross-sectional study

**DOI:** 10.1186/s12879-021-06087-7

**Published:** 2021-04-26

**Authors:** Sophaphan Intahphuak, Tawatchai Apidechkul, Patita Kuipiaphum

**Affiliations:** 1 School of Nursing, Mae Fah Luang University, Chiang Rai, 57100 Thailand; 2 School of Health Science, Mae Fah Laung University, Chiang Rai, 57100 Thailand; 3 Mae Je Dee Mai Sub-District Health Promotion Hospital, Wiang Pa Pao, Chiang Rai, 57260 Thailand

**Keywords:** Antibiotic resistance, Prevalence, Lahu, Hill tribe, Thailand

## Abstract

**Background:**

Antibiotic resistance is often reported and great concerned as one of public health problems especially people living with poverty in developing countries including Thailand. The hill tribe people is defined as vulnerable population for antibiotic resistance in Thailand due to poor economic and education status particularly the Lahu people who is the second greatest group of the hill tribe people in Thailand. The study aimed to estimate the prevalence, factors associated with, and typing major species of bacteria with antibiotic drugs resistance among the Lahu hill tribe people in northern Thailand.

**Methods:**

A cross-sectional study was conducted to gather the information from the participants. A validated questionnaire was used for data collection. Participants who presented an illness related to infectious diseases were eligible to participate the study and were asked to obtain specific specimen; sputum, urine or stool. Antibiotic *susceptibility* was tested by Kirbey Bauer’s disc diffusion test. Chi-square and logistic regression were used to detect the associations between variables at the significant level of α = 0.05.

**Results:**

A total of 240 participants were recruited into the study. The majority had urinary tract infection (67.9%) with two major pathogenic species of the infection*; Escherichia coli* (12.8%), and *Enterobacter cloacae* (8.0%). The prevalence of antibiotic resistance was 16.0%. *Escherichia coli and Klebsiella pneumoniae* species were found to have multidrug resistance that was greater than that of other species, while ampicillin was found to have the greatest drug resistance. It was found that those who had poor knowledge of antibiotic use had a 2.56-fold greater chance (95% CI = 1.09–5.32) of having antibiotic resistance than did those who had good knowledge of antibiotic use, and those who had poor antibiotic use behaviors had a 1.79-fold greater chance (95% CI = 1.06–4.80) of having antibiotic resistance than did those who had good antibiotic use behaviors.

**Conclusion:**

Effective public health interventions are urgently needed to reduce antibiotic drug resistance among the Lahu people by improving their knowledge and skills regarding the proper use of antibiotics and eventually minimizing antibiotic resistance. Moreover, health care professionals should strictly follow the standard guideline to prescribe antibiotics.

## Background

Antibiotic resistance is a recently emerging global threat to human health [1]. Some antibiotic drugs become ineffective for a particular treatment and increase all medical expenses, from longer stays at a health institute to the use of drugs. As a result, the morbidity and mortality rates attributable to this problem have been rising every year [1, 2]. The World Health Organization (WHO) has also reported that some kinds of human diseases have become unresponsive to treatment, such as pneumonia, tuberculosis, gonorrhea, and salmonella. Antibiotic resistance is also caused by misuse and by an accelerated food production process, particularly that of animal products [1]. People in developing countries are more vulnerable to antibiotic resistance than are those living in developed countries due to poor education and poor access to standard medical services [3]. To date, the problem of antibiotic resistance does not persist only in health institutes, but it is also present in people living in a community, especially in a community located far away from the city and among people with a poor educational level and economic status [4]. The World Health Organization (WHO) reported that the major causes of antibiotic resistance were accelerated by the misuse and overuse of antibiotics, as well as poor infection prevention and control [1].

The Ministry of Public Health in Thailand [5] reported that at least 88,000 antibiotic resistance cases were reported each year, and 40.0% died. Moreover, more than 40,000 million Thai baht, or 0.6% of the GDP, were spent to address this problem. In 2016, Thailand launched a 5-year national strategic plan to reduce antimicrobial resistance (NSP-AMR:2017–2021) aimed at reducing antibiotic misuse by enhancing public awareness and reducing antibiotic resistance in the Thai population [6, 7]. Commonly, Thai people are able to access antibiotics without prescriptions, such as through buying drugs from private drugstores or pharmacies, including from a small grocery in a community [8]. Moreover, self-medication is a common health behavior in Thai individuals [9]. Pumtong et al. [10] reported that a lack of knowledge and misunderstandings about antibiotics were important factors in the misuse of antibiotics among Thai people, particularly those living in remote areas.

Chiang Rai Province is located in northern Thailand and has long borders with neighboring countries such as Myanmar and Laos PDR. Many groups of hill tribe people live along the borders. Most hill tribe people have migrated from South China over many decades to settle in the remote and mountainous areas in northern Thailand. The hill tribe in Thailand has six main groups: Akah, Lahu, Homh, Yao, Karen, and Lisu [11]. The Lahu is the second-largest group among the hill tribe people in Thailand [12]; they usually live in the highlands [13]. The Lahu people have their own culture, language and lifestyle [13] and have very low literacy due to limited access to Thailand’s educational system. Given their low family economic status, the Lahu people do not have many choices for access to medicines when they experience health problems. Buying drugs, especially antibiotics, from a small grocery in a community is the most familiar and available option for them [14].

Thus, this study aimed to estimate the prevalence of and factors associated with major species of bacteria resisting antibiotics in the Lahu hill tribe people in northern Thailand.

## Methods

### Study design

A cross-sectional study was applied to gather the information from the participants.

### Study setting

The study settings were Lahu villages from Wiang Pa Pao District, Chiang Rai Province, Thailand. The study settings are located away from the center of Chiang Rai Province with 126 km.

There were three selected villages for the study with total 1865 people living in villages. The villages are located in the rural area with seasoning poor transportation especially in the rainy season, then they are facing a difficulty in assessing health service. A large number of diarrheas were reported in these villages previous years through health promoting hospital system, so called Thailand Health Data Center System which is used in the whole country of Thailand for report the infectious diseases [15]. The locations are approximately 42 km far from Wiang Pa Pao District, Chiang Rai Province, Thailand. A health center is located average 12 km far from the villages, and 28 km from the health center to a district hospital with 30 min by car. Moreover, it needs an hour from the district hospital to the provincial hospital (tertiary hospital) by car.

The health center is responsible for 14 villages with the total population at 9632 (4815 males, 4817 females). There are 9 public health staffs; two nurses, two public health professional, 5 other health alien professionals. Three different antibiotics were available at health center; amoxicillin, roxithromycin, and dicloxacillin. Top five infection diseases reported from health center between January and December 2019 were 1) diarrhea, 2) pyrexia, 3) dengue fever, 4) conjunctivitis, and 5) hand, foot and mouth disease [16]. Top three of infectious diseases among the people living in selected villages were 1) diarrhea, 2) common cold, and 3) cystitis [16].

### Study population

The Lahu people aged 15 years and over and living at three selected villages, and presenting at least one of signs or symptoms of an infection during the data collection date were the study population.

### Inclusion and exclusion criteria

The Lahu hill tribe people who had signs and symptoms of respiratory tract system, gastrointestinal tract system, or urinary tract infection during the data collection date were met the inclusion criteria of the study. The information on signs and symptoms relevant to the infection of these systems were also provided to village headmen to use it as preliminary criteria such as having fever, cough headach for respiratory tract infection between 1 week prior to the date of data collection. Having diarrhea was used as the major sing of gastrointestinal tract system, and dysuria, suprabic pain were used to identify the urinary tract infection one-week prior to the date of data collection. Those who had respiratory tract infection, they were asked to obtain suptum. Stool sample were asked for those who had an illness related to gastrointestinal tract system. While urine sample were asked from those who had an illness related to urinary tract infection. Those who could not provide essentail information regarding the study protocol were excluded from the study. All participants were asked to collect a suitable specimen (stool, sputum, urine) according to their illness presentation.

### Study sample

The standard formula sample size calculation for a cross-sectional study was used to estimate the sample size [17], *n* = Z^2^_α/2_P(1-P)/e^2^, where Z is the standard normal distribution at the confident 95%, then the Z = 1.96; P is the estimate true proportion which was estimated by the data from previous study, 23.0% [18]; and the e is the accepted deviation, 0.055, then 224 participants were needed. Adding for 5.0% for any error in the process of the study, 235 participants were required for the analysis.

### Materials and measurements

A questionnaire was developed from the literature review regarding the behaviors of antibiotic use and laboratory testing on antibiotic resistance. The questionnaire consisted of seven parts. In part one, 6 questions were used to collect sociodemographic characteristics, such as sex, age, highest education level, and marital status. In part two, 9 questions were used to collect data on the experiences of antibiotic use within the past 12 months, such as the source of antibiotic assessment, frequency, reason for taking antibiotics, and source of antibiotic knowledge. In part three, 10 questions were used to collect knowledge on infectious diseases. Those who scored 60.0% and over were defined as having good knowledge [19, 20]. Those who had scores of 59.0% and lower were defined as having poor knowledge. In part four, 10 questions were used to assess knowledge of antibiotic use in daily life. Those who scored 60.0% and over were defined as having good knowledge. Those who had scores of 59.0% and lower were defined as having poor knowledge. In part five, 10 questions were used to detect attitudes on antibiotic use. Those who scored 60.0% and over were defined as having a good attitude. Those who had scores of 59.0% and lower were defined as having a poor attitude. In part six, 25 questions were used to collect information on antibiotic use behaviors. The last part (part 7) was used for collecting village information, including basic health information from the heath center in the community.

The quality of the questionnaire was validated by three steps. First, the content was validated by the item-objective congruence (IOC) method by five relevant experts: two medical doctors, an epidemiologist, a pharmacist, and a community nurse. The experts provided comments on the questions and a score. Questions that were scored less than 0.5 were excluded from the questionnaire set, those that were scored 0.5–0.7 were revised according to the comments, and questions that were scored greater than 0.7 were included in the questionnaire set. In the second step, a pilot was executed to detect the reliability and feasibility of the questionnaire among 15 samples who had characteristics similar to those of the study samples in Mae Suai District (district near Wiang Pa Pao District), Chiang Rai Province, Thailand. The overall reliability of parts three, four, and five were found to be 0.77, 0.78, and 0.78, respectively. In the last step, the questionnaire was revised and piloted again for 15 additional participants who had characteristics similar to those of the study samples.

All participants were asked to collect suitable specimens according to their illness. Each individual was asked for one specimen. All specimens were transferred to the Men Rai Laboratory Center, Chiang Rai, Thailand, on the same day. The Kirbey-Bauer disc diffusion test, based on the Clinical and Laboratory Standards Institute (CLSI) Guidelines, was used to detect antibiotic resistance.

### Study procedure

Permission to access villages was granted by the district public health officer. Village headmen were contacted 3 days before having a small meeting. All information regarding the study was provided to the village headmen and village health volunteers. Before the date of data collection, all village members who met the eligible criteria were informed about the study protocols and invited to participate in the study. At the date of data collection, all participants were asked to obtain an informed consent form before data collection. For participants younger than 18 years old, informed consent forms were also obtained from their parents before data collection. The questionnaire was completed with the help of 15 village health volunteers (five persons in each village) who were fluent both the Thai and Lahu languages. They were trained for 6 hours on the content of the study, including a questionnaire, to clarify their understanding of and familiarize them with the full content of the questionnaire. The interviews lasted 25 min in each. Specimens were collected by licensed medical technicians from a hospital. Participants’ information such as name, ID number, specimen type and date of collection were labeled in individuals’ container. The sputum collection, participants were asked to collect their sputum by expectorating deep cough sputum directly into a sterile dry container. For urine collection, participants were asked to collect midstream urine with 10–20 mL in a clean and dry container. For the stool collection, participants were asked to collected 10–20 mL sample and put into a clean and dry container [21].

### Laboratory work

All 240 participants were asked to collect specimens; 61 participants had sputum collected, 173 participants had urine collected, and 6 participants had stool collected. Specimens were kept and transferred for laboratory work at Chiang Rai city on the same day. Different specimens were placed on a specific plate before incubation at 35 °C for 24 h. Afterwards, the colonies were observed for the detection of pathogenic typing. Sensitivity to antibiotics was detected by the Kirbey-Bauer disc diffusion test to determine antibiotic resistance. The methods in laboratory were done under The Clinical and Laboratory Standards Institute (CLSI) Guidelines.

The laboratory test was reported in three forms: susceptible (S), intermediate (I), and resistance (R).

### Statistical analysis

Data were double-entered into Excel spreadsheets and were checked for any errors before being transferred into SPSS version 24 (SPSS, Chicago, IL) for further analysis. Descriptive statistics were used to describe the participants’ characteristics in both categorical and continuous data. Chi-square tests and logistic regressions were used to identify the factors associated with antibiotic resistance among the participants at the significance level of α = 0.05. In the logistic regression steps, the Hosmer-Lemeshow chi-square test was used to assess the overall model prediction before and after the variables were included into the model. The fitness of the model was determined by the Cox-Snell R^2^ and Nagelkerke R^2^ before the results were interpreted.

Classification of variables in the analysis, age was classified into two groups: < 60 years, ≥ 60 years. Antibiotic resistance was classified into two groups: resistance and non-resistance. Those who were susceptible and intermediate were classified as non-resistance, while resistance was classified as resistance in the analysis.

## Results

A total of 240 participants participated the study; 70.4% were female, 25.4% were aged 30-40 years and 82.9% were married. More than half worked in the agricultural sector (55.4%) and had poor education, which was less than primary school (45.8%) (Table 1).

**Table 1:** General characteristics of participants

Characteristic	n	%
**Total**	240	100.0
**Sex**		
Male	71	29.6
Female	169	70.4
**Age** (years)		
10–20	10	4.2
21–30	40	16.7
30–40	61	25.4
41–50	59	24.6
51–60	22	9.2
61–70	35	14.6
> 70	13	5.4
Mean = 43.5, SD = 15.3, Min = 17, Max = 98		
**Education**		
No education	110	45.8
Primary school	110	45.8
High school	14	5.8
Vocational certificate and higher	6	2.5
**Occupation**		
Unemployed	23	9.6
Agriculturalist worker	133	55.4
Employee	67	29.9
Commercial	17	7.1
**Married status**		
Single	20	8.3
Married	199	82.9
Ever married	21	8.8
**Underlying disease**^**a**^		
No	193	80.4
Yes ^a^	47	19.6
Hypertension (*n* = 47)	23	
Gastritis(*n* = 47)	13	
Diabetes mellitus(*n* = 47)	12	
Chronic renal failure(*n* = 47)	4	
Asthma(*n* = 47)	3	
Coronary heart disease(*n* = 47)	2	
**Illness and specimen**		
Urinary tract infection (Urine)	173	72.1
Respiratory tract infection (Sputum)	61	25.4
Gastrointestinal tract infection (Stool)	6	2.5

^a^ Some participants had more than one list of a disease

Urinary tract infectious diseases accounted for the largest group of infections in the past 12 months (67.9%), with 163 cases of urinary tract infection. A community health center (28.8%) was the favored location of healthcare services for the participants, followed by a district hospital (24.6%) and a drugstore (21.7%). The top three sources of antibiotic use were district hospitals (51.7%), drugstores (27.9%), and private medical clinics (22.5%).More than half of the participants received information about infectious diseases and antibiotics from health care professionals (Table 2).

**Table 2:** History of illness, and access medical services

Factors	n	%
**Total**	240	100.0
**History of illness one-year prior**^a^		
Urinary tract infection (*n* = 240)	163	67.9
Respiratory tract infection (*n* = 240)	135	56.3
Gastrointestinal tract infection (*n* = 240)	81	33.8
**First priority on access a medical care while having health problem**		
Community health center	69	28.8
District hospital	59	24.6
Drugstore	52	21.7
Village grocery	39	16.3
Private clinic	13	5.4
Using leftover drugs	4	1.7
Using drugs from family member	3	1.3
**Frequency of antibiotic use 12 months prior** (times)		
1	153	63.8
2	58	24.2
≥ 3	29	12.1
**Source of getting antibiotics**^a^		
District hospital (*n* = 240)	124	51.7
Drugstore (*n* = 240)	67	27.9
Private medical clinic (*n* = 240)	54	22.5
Private nurse clinic (*n* = 240)	38	15.8
Health center (*n* = 240)	16	6.7
Tertiary hospital (*n* = 240)	10	4.2
Family member or neighborhood (*n* = 240)	2	0.8
**Reasons for taking antibiotic**^a^		
Wish to get better from an illness (*n* = 240)	167	69.6
Doctor or health care professional prescribed (*n* = 240)	151	62.9
Protect severity of illness (*n* = 240)	77	32.1
**Ever receiving information on infectious diseases**		
No	64	26.7
Yes	176	73.3
**Source of information about infectious disease** ^a^		
Health care professional (*n* = 240)	129	53.8
Village health volunteer (*n* = 240)	74	30.8
Community leader (*n* = 240)	70	29.2
Television, radio (*n* = 240)	66	25.5
**Received information on antibiotic**		
No	62	25.8
Yes	178	74.2
**Source of information about antibiotic**^a^		
Health care professional (*n* = 240)	150	62.5
Village health volunteer (*n* = 240)	83	34.6
Community leader announce (*n* = 240)	76	31.7
Television or radio broadcasting (*n* = 240)	62	25.8

^a^ Participants provided more than a choice

Most of these sources showed good knowledge of infectious diseases (82.9%), while knowledge of and attitudes toward antibiotics and practices were poor (66.2, 51.2, and 64.6%, respectively) (Table 3).

**Table 3:** Knowledge on infectious diseases, and knowledge, attitudes, and behaviors regarding antibiotic use

Items	n	%
**Knowledge on infectious diseases**		
Poor	41	17.1
Good	199	82.9
**Knowledge regarding antibiotic use**		
Poor	159	66.2
Good	81	33.8
**Attitudes regarding antibiotic use**		
Poor	123	51.2
Good	117	48.8
**Behavior regarding antibiotic use**		
Poor	155	64.6
Good	85	35.4

According the comparisons between those who had drug resistance and those who did not, only knowledge regarding antibiotic use was found to be statistically significant (*p*-value = 0.034). However, two other variables (age and antibiotic use behaviors) were found to have marginally significant differences between groups (Table 4).

**Table 4:** Factors correlation with antibiotic resistance

Factors	Resistance		No resistance		χ^**2**^	***p***-value
	n	%	n	%		
**Total**	**30**	**12.5**	**210**	**87.5**	**N/A**	**N/A**
**Age** (years)						
< 60	20	66.7	172	81.9	3.81	0.051
≥ 60	10	33.3	38	18.1		
**Education**						
No	18	60.0	92	43.8	2.77	0.096
Yes	12	40.0	118	56.2		
**Receiving antibiotic information**						
No	8	26.7	54	25.7	0.01	0.911
Yes	22	73.3	156	74.3		
**Knowledge on infectious diseases**						
Poor	6	20.0	35	16.7	0.20	0.650
Good	24	80.0	175	83.3		
**Knowledge regarding antibiotic use**						
Poor	25	83.3	134	63.8	4.47	0.034*
Good	5	16.7	76	36.2		
**Attitudes regarding antibiotic use**						
Poor	19	63.3	104	49.5	2.00	0.157
Good	11	36.7	106	50.5		
**Behaviors on antibiotic use**						
Poor	24	80.0	131	62.4	3.56	0.059
Good	6	20.0	79	37.6		
**Frequency of antibiotic use 12 months prior** (time)						
1	22	73.3	131	62.4	1.36	0.243
≥ 2	8	26.7	79	37.6		

* Significant level at α = 0.05

Of 240 specimens (participants), 187 samples were found to contain bacterial species, including normal flora. Fifty-five specimens were found to have more than 3 bacterial species, and only 30 samples were found to have drug resistance. The top-five bacterial species were detected in the specimens: *Escherichia coli* (12.8%*), Enterobacter cloacae* (8.0%), *Klebsiella pneumoniae* (4.3%), *Enterococcus faecalis* (4.3%), and *Staphylococcus coagulase negative* (4.3%). The prevalence of antibiotic resistance was 16.0%. *Klebsiella pneumoniae* resisted ampicillin, cefazolin, cefoxitin, ciprofloxacin, levofloxacin and trimetroprim-sulfamethoxazole (TMP-SMX), while *Escherichia coli* demonstrated resistance to ampicillin, amoxicillin/clavulanic acid (amoxy-clav®), gentamycin, ciprofloxacin, levofloxacin and TMP-SMX. Ampicillin was found to have the highest resistance rate among the pathogenic bacteria, followed by cefoxitin, cefazolin, amoxy-clav®, and TMP-SMX (Table 5).

**Table 5:** Types of bacterial infection and antibiotic resistance (*n* = 30)

Antibiotic	Amount of infection	Ampicillin	TMP-SMX	Penicillin	Amoxicillin /Clavu lanic acid	Cefoxitin	Cefazolin	Doxy cycline	Gentamycin	Cipro foxacin	Levofloxacin	Vancomycin	Oxa cillin	Methicillin
Bacterial species														
*Escherichia coli*	7	6	6		1				2	1	1			
*Enterobacter cloacae*	3	4			4	4	1							
*Klebsiella pneumoniae*	7	7	1			1	1			1	1			
*Staphylococcus coagulase negative*	3			3				1					1	1
*Salmonella spp.*	1	1	1	1						1	1			
*Citrobactor Freundii*	1	2			1	1	1							
*Enterococcus faecalis*	3							3	1					
*Staphylococcus aureus*	2			2				1				1		
*Klebsiella aerogenes*	1	1			1	1	1							
*Proteus mirabilis*	1	1	1	1					1					
*Streptococus agalactiae*	1	1												
*Citrobacter koseri*	1	1												
*Klebsiella oxytoca*	1	1												
*Total*		25	9	7	7	7	4	4	4	3	3	1	1	1

Notation: 1 person could infect more than one type bacterial species and antibiotics

Two variables were found to be associated with antibiotic drug resistance among the Lahu people: knowledge and behaviors regarding antibiotic drug resistance. In the multivariate model, these two variables remained associated with antibiotic resistance, including knowledge and behaviors regarding antibiotic resistance. Those who had poor knowledge reading antibiotic use had a 2.56-fold greater chance (95% CI = 1.09–5.32) of having antibiotic resistance than did those who had good knowledge of antibiotic use. Those who had poor antibiotic use behaviors had a 1.79-fold greater chance (95% CI = 1.06–4.80) of having antibiotic resistance than did those who had good antibiotic use behaviors (Table 6).

**Table 6:** Factors associated with antibiotic resistance in univariate and multivariate analyses

Factors	Drug-resistance		No drug-resistance		Univariate analyses			Multivariate analyses		
	n	%	n	%	OR	95% CI	***p***-value	AOR	95% CI	***p***-value
**Total**	30	12.5	210	87.5						
**Age** (years)										
< 60	20	66.6	172	81.9	1.00					
≥ 60	10	33.3	38	18.1	2.26	0.98–5.22	0.056			
**Education**										
No	18	60.0	92	43.8	1.92	0.88–4.19	0.100			
Yes	12	40.0	118	56.2	1.00					
**Received antibiotic information**										
No	8	26.7	54	25.7	1.05	0.44–2.49	0.911			
Yes	22	73.3	156	74.3	1.00					
**Knowledge on infectious diseases**										
Poor	6	20.0	35	16.7	1.25	0.48–3.28	0.651			
Good	24	80.0	175	83.3	1.00					
**Knowledge regarding antibiotic use**										
Poor	25	83.3	134	63.81	2.80	1.04–7.71	0.041*	2.56	1.09–5.32	0.048*
Good	5	16.7	76	36.2	1.00			1.00		
**Attitudes regarding antibiotic use**										
Poor	19	63.3	104	49.5	1.76	0.80–3.88	0.161			
Good	11	36.7	106	50.5	1.00					
**Behavior regarding antibiotic use**										
Poor	24	80.0	131	62.4	2.41	1.02–6.16	0.046*	1.79	1.06–4.80	0.041*
Good	6	20.0	79	37.6	1.00			1.00		
**Frequency of antibiotic use last 12 month** (time)										
1	22	73.3	131	62.4	1.00					
≥ 2	8	26.7	79	37.6	0.60	0.25–1.41	0.24			

* Significant level at α = 0.05

## Discussion

The Lahu villages were located far away from a health center while the Lahu people lived with low education and poor family economic. They were facing several infectious diseases which were required a treatment by antibiotic. A few lists of antibiotic drugs were available in community health center where trained health professionals were working. Almost one-third of the Lahu people were practicing self-prescription by buying drugs from private pharmacy or village grocery. Urinary tract infection was reported in the greatest portion of infectious diseases among the Lahu people. Two-thirds were having poor knowledge and poor behaviors on antibiotic drugs use, and more than half had a poor attitude towards antibiotic use. The prevalence of antibiotic resistance was 16.0%. The *Klebsiella pneumoniae* showed resistant to ampicillin, cefazolin, cefoxitin, ciprofloxacin, levofloxacin and TMP-SMX while *Escherichia coli* demonstrated resistance to ampicillin, amoxy-clav®, gentamycin, ciprofloxacin, levofloxacin and TMP-SMX. Ampicillin was found the highest resistance rate among the pathogenic bacteria flowing by cefoxitin, cefazolin, amoxy-clav®, and TMP-SMX. A poor knowledge and behaviors reading antibiotic drug were associated with having antibiotic drug resistance among the Lahu people.

In our study, the prevalence of antibiotic resistance was 16.0%, and bacteria from urinary tract infections were the major cause of antibiotic resistance. A report from the Ministry of Public Health in Thailand said that the five most common bacterial species involved in antibiotic resistance were *Escherichia coli (E. coli), Klebsiella pneumoniae (K. pneumoniae), Acinetobacter baumannii (A. baumannii), Pseudomonas aeruginosa (P. aeruginosa)* and methicillin-resistant *Staphylococcus aureus* (MRSA) [5]. A study in southern region of Thailand was reported that the resistance rate for amoxicillin, doxycycline and TMP-SMX were 53.3, 51.3, and 24.0%, respectively [22]. A community study in Tanzania [23] found that *Escherichia coli* strains were mostly present in infections and antibiotic resistance. In addition, a hospital-based study in Lebanon [24] reported that the frequency of antibiotic resistance was 53.7% (39.9% were multidrug-resistant), and different kinds of pathogenic bacteria that were resistant to different kinds of antibiotics, such as *Escherichia coli* strains, were mostly susceptible to carbapenems and tigecycline, while *Klebsiella* species were mostly susceptible to amikacin and carbapenems. Moreover, a study in Hungary showed that the frequency of prescriptions from clinicians was related to the percentage of antibiotic resistance [25]. In addition, a prospective cohort study demonstrated that several bacterial pathogenic species were resistant to different kinds of antibiotics in the United Kingdom; 43.0% of patients with *E. coli* were resistant to at least one antibiotic, with the highest resistance to amoxicillin [26]. This reflects that the hill tribe people in Thailand are facing in a lower rate of antibiotic resistance while compared to other populations. However, with the condition of a few studies of a community-based antibiotic resistance in Thailand, they may need to have more research findings before able to make an accurate conclusion.

A study in Kenya [27] showed that several bacteria causing diarrhea were resistant to different kinds of antibiotics, and the misuse and improper dosing of drugs were the major factors contributing to antibiotic resistance. Moreover, a study in the rural hill communities of northeastern India [28] found that several antibiotics had high resistance rates, such as ampicillin (92.0%), ceftazidime (90.0%), and tetracycline (36.0%). The study also reported that the education of participants was strongly associated with antibiotic resistance.

A study on the epidemiology of antibiotic resistance in some selected communities in Thailand found that pathogeneses from upper respiratory tract and digestive tract infections were most common in antibiotic drug resistance [29]. A systemic review of antimicrobial resistance among pathogenic bacteria in Southeast Asia reported pathogenic species resistance to penicillin among the pathogeneses causing respiratory and digestive tract infections [30], which are findings similar to those in our study.

In our findings, we also found that knowledge and misuse antibiotic behaviors were associated with antibiotic resistance among the Lahu hill tribe people. This was supported by a study by Castro-Sánchez et al. [31], which reported that the major significant factor for antibiotic resistance was the misuse of antibiotics in both humans and animals. Moreover, a 5-year analysis on antibiotic resistance was reported even in high-standard hospitals, with several antibiotic resistances found particularly among patients who had a history of misusing antibiotics prior to being admitted to the hospital, especially those who had poor knowledge of antibiotic use [32]. Additionally, a study in Thailand reported that people who had higher educational levels and those with greater wealth were associated with having antibiotic drug resistance [8].

The results from this study confirm that the problem of antibiotic resistance extends into the community, even in remote areas. It reflects a need for the design of an effective strategy at the nationwide level to address the problem, particularly among those who have poor economic and educational status. Moreover, improving access to health centers to obtain antibiotics while requiring their careful and proper use for the treatment of people living in remote areas is critical in Thailand.

Some limitations were found during the study. First, some participant could not use Thai effectively, then we asked the village health volunteer who fluent both Thai and Lahu languages to help in completion the questionnaire and collecting specimen. However, some questions particularly in asking the questions about the urinary tract infection in females, the outcome might not be accurate and could impact the findings. Second, asking question on experience in use of antibiotics one year prior, it is one of our concerns during the study. Participants were asked and confirmed their response before responding to the questions. Third, knowing on the type of antibiotics, all antibiotic drugs available in village grocery and health center were shown to ensure that participants were able to identify the antibiotics. Last, generalization the findings to other populations would be limited due to the study participants were selected from three villages with high incident of the diseases.

## Conclusion

The Lahu people in Thailand are facing major challenges in their access to standard medical care due to the location of their village, which is far from the city and health center. They are also improperly using antibiotics, which had led them to have several bacterial infections that have eventually become resistant to antibiotics. Majority of the Lahu people receive antibiotics from the health care facility. Poor education and poor behaviors regarding the use of antibiotics are major factors influencing antibiotic resistance among the Lahu people. Public health interventions for improving knowledge and practices regarding the use of antibiotics should be urgently implemented. A regulation to prevent untrained health professionals from selling antibiotics, particularly in small groceries in villages, should be developed and implemented to reduce the improper use of antibiotics and minimize antibiotic resistance among the Lahu people in Thailand. Moreover, antibiotic prescription among health professionals should also be seriously followed the standard guideline. The surveillance of antibiotics resistance system should be immediately implemented and used as a major tool for monitoring antibiotics resistance in a whole country.

## Data Availability

The raw data available upon reasonable request from the corresponding author.

## Abbreviations

CLSI: Clinical and Laboratory Standards Institute
IOC: Item-objective congruence
GDP: Gross domestic product
TMP-SMX: Trimethoprim-Sulfamethoxazole
WHO: World Health Organization
